# Effects of cinnamaldehyde on anti-respiratory syncytial virus: A protocol of systematic review and meta-analysis

**DOI:** 10.1097/MD.0000000000020266

**Published:** 2020-05-15

**Authors:** Lan Feng, Jing Li, Hai-Bo Yu, Qing Xue, Li-Juan Dai

**Affiliations:** a Department of Infectious Diseases, First Affiliated Hospital of Jiamusi University; b Department of Physiology, Jiamusi University School of Basic Medical Sciences; c Department of Cardiology, First Affiliated Hospital of Jiamusi University; d Clinical Medicine of Class 7 in Grade 2016, Jiamusi University, Jiamusi, China.

**Keywords:** anti-respiratory syncytial virus, cinnamaldehyde, effect

## Abstract

**Background:**

Previous reports found that cinnamaldehyde has effects on anti-respiratory syncytial virus (ARSV). However, their results are still contradictory. Therefore, this study will systematically address the effects of cinnamaldehyde on ARSV.

**Methods:**

The following electronic bibliographic databases will be retrieved from their outset to the March 31, 2020: MEDLINE, EMBASE, Cochrane Library, Cumulative Index to Nursing and Allied Health Literature, Technology Periodical Database, China Biology Medicine, and China National Knowledge Infrastructure. No language and publication time limitations will be exerted in this study. All relevant case-controlled studies or randomized controlled studies exploring the effects of cinnamaldehyde on ARSV will be included. Study quality of case-controlled studies will be assessed by Newcastle–Ottawa scale, and that of randomized controlled studies will be identified by Cochrane risk of bias tool. All data pooling and analysis will be performed using RevMan 5.3 software.

**Results:**

This study will summarize the up-to-date high-quality evidence to synthesize outcome data on the effects of cinnamaldehyde on ARSV.

**Conclusion:**

Findings of this study may provide beneficial evidence for both clinicians and future studies regarding the effects of cinnamaldehyde on ARSV.

**Systematic review registration:**

INPLASY202040074.

## 1 Introduction

Respiratory syncytial virus is one of the most leading causes that lead to lower respiratory tract disease in infants and young children.^[1,2,3,4,5]^ It mostly occurs in infants of 2 to 3 months old with high morbidity and mortality.^[6,7,8]^ The major risk factors are prematurity, bronchopulmonary dysplasia, and congenital heart disease.^[9,10]^ Previous study reported that it accounts for more than 75% of all childhood bronchiolitis, and 40% of all children pneumonias.^[11]^ Thus, anti-respiratory syncytial virus (ARSV) intervention is very urgent to manage such condition.^[12,13,14,15,16]^ Several studies have reported that cinnamaldehyde has effects on ARSV.^[17,18,19,20]^ However, there is a lack of supportive evidence on the effects of cinnamaldehyde on ARSV. Therefore, this study aims to conduct a systematic review and meta-analysis of case-controlled studies (CCSs) or randomized controlled studies (RCSs) on the effects of cinnamaldehyde on ARSV.

## 2 Methods

### 2.1 Study registration

This study has been registered on INPLASY202040074. It has been conducted according to the preferred reporting items for systematic reviews and meta-analysis protocol statement guidelines.^[21,22]^

### 2.2 Eligibility criteria

#### 2.2.1 Types of trials

We will only include potential CCSs or RCSs exploring the effects of cinnamaldehyde on ARSV.

#### 2.2.2 Types of subjects

This study will include respiratory syncytial virus infects host HeLa cells as its research targets.

#### 2.2.3 Types of interventions

In the experimental group, all studies utilized cinnamaldehyde alone for the treatment of ARSV.

In the control group, studies used any comparators will be included, such as no treatment. However, we will not include studies using cinnamaldehyde.

#### 2.2.4 Types of outcome measurements

Primary outcome is apoptotic host HeLa cells, as detected by flow cytometry.

Secondary outcomes are apoptosis-related proteins expression, as measured by immunofluorescence or western blot test. These proteins include Caspase-3, Caspase-9, p-AKT, Bcl-2, and Bax.

### 2.3 Information sources and search strategy

This study will search MEDLINE, EMBASE, Cochrane Library, Cumulative Index to Nursing and Allied Health Literature, Technology Periodical Database, China Biology Medicine, and China National Knowledge Infrastructure from their outset to the March 31, 2020 without restrictions of language and publication time. We will consider all potential CCSs or RCSs that examined the effects of cinnamaldehyde on ARSV for inclusion. A detailed search strategy for MEDLINE is summarized (Table 1). Identical search strategies with specifics will be adapted and applied to the other electronic databases. We will also search other resources, such as relevant conference proceedings, and reference lists of included studies.

**Table 1:**
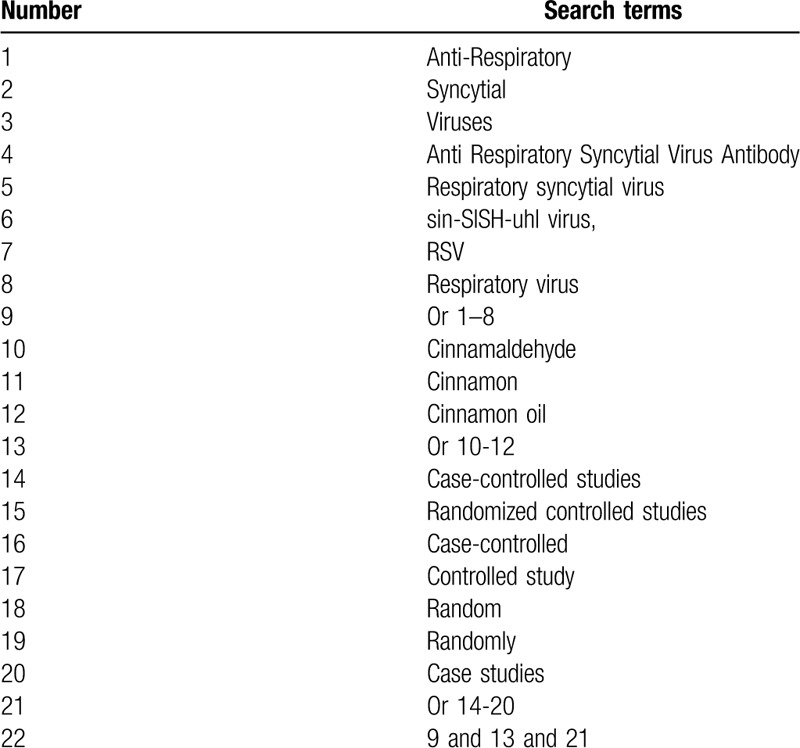
Detailed search strategy of MEDLINE.

### 2.4 Study selection

Two researchers will independently identify studies for inclusion in 2 steps. At the first step, we will examine the titles and abstracts for potentially relevant citations; and duplicates and unrelated studies will be eliminated. At the second step, we will obtain full-text of potential papers and will carefully read them based on the all eligibility criteria. Divergences will be solved by discussion with a third researcher involved. Details of entire study selection process will be presented in a preferred reporting items for systematic reviews and meta-analysis flow diagram.

### 2.5 Data extraction and management

Two researchers will independently extract data using a previous designed data extraction template. It consists of study characteristics (title, first author, journal, etc), information of targeted Host cell Hela, study design (types of study, sample size, etc), intervention and control details (types of treatments, dosage, etc), outcomes, results, findings, and other related information. In case of unclear or missing data, we will contact primary authors to obtain it. Any uncertainties will be solved by discussion with a third researcher involved.

### 2.6 Study quality assessment

Two researchers will independently assess study quality for each included study. The study quality of CCSs will be evaluated by Newcastle–Ottawa scale, and study quality of RCSs will be appraised by the Cochrane risk of bias tool. Any divergences will be resolved by discussion with the help of a third researchers invited.

### 2.7 Statistical analysis

Statistical analyses will be undertaken by RevMan 5.3 software. We will use risk ratio and 95% confidence intervals to calculate dichotomous data, and will utilize mean difference or standardized mean difference and 95% confidence intervals to present continuous data. We will check heterogeneity using *I*^*2*^ test. *I*^*2*^ ≤ 50% means homogeneity and fixed-effects model will be employed. If possible, we will conduct a meta-analysis. *I*^*2*^ > 50% indicates remarkable heterogeneity, and we will utilize a random-effects model. In addition, we will perform a subgroup analysis to examine the possible sources of obvious heterogeneity.

### 2.8 Additional analysis

#### 2.8.1 Subgroup analysis

We will perform a subgroup analysis based on the types of studies, different intervention and controls, and outcomes.

#### 2.8.2 Sensitivity analysis

We will carry out a sensitivity analysis to test the stability of study findings by removing low methodological quality studies.

#### 2.8.3 Reporting bias

All reporting bias will be examined by a funnel plot and Egger regression test if over 10 eligible studies are included.

### 2.9 Dissemination and ethics

This study will not employ any individual patient information, thus, no ethical approval is needed. We will plan to publish this study on a peer-reviewed journal or conference presentation.

## 3 Discussion

Published studies reported the effects of cinnamaldehyde on ARSV. However, there is not a systematic review to explore this issue. Thus, this study will firstly provide concise literature sources of the present evidence of cinnamaldehyde on the effect of ARSV. Its results will supply helpful reference for clinicians, policy makers, stakeholders, and researchers. In addition, its findings may help to investigate the research gaps and opportunities for the future researchers.

## Author contributions

**Conceptualization:** Lan Feng, Jing Li, Hai-Bo Yu, Qing Xue, Li-Juan Dai.

**Data curation:** Lan Feng, Jing Li, Li-Juan Dai.

**Formal analysis:** Lan Feng, Jing Li, Hai-Bo Yu, Qing Xue.

**Funding acquisition:** Jing Li, Li-Juan Dai.

**Investigation:** Jing Li, Li-Juan Dai.

**Methodology:** Lan Feng, Hai-Bo Yu, Qing Xue.

**Project administration:** Jing Li, Li-Juan Dai.

**Resources:** Lan Feng, Hai-Bo Yu, Qing Xue.

**Software:** Lan Feng, Hai-Bo Yu, Qing Xue.

**Supervision:** Jing Li, Li-Juan Dai.

**Validation:** Lan Feng, Jing Li, Hai-Bo Yu, Qing Xue, Li-Juan Dai.

**Visualization:** Lan Feng, Jing Li, Li-Juan Dai.

**Writing – original draft:** Lan Feng, Jing Li, Qing Xue, Li-Juan Dai.

**Writing – review and editing:** Lan Feng, Jing Li, Hai-Bo Yu, Qing Xue, Li-Juan Dai.
